# Circulating Brain-Derived Neurotrophic Factor and Indices of Metabolic and Cardiovascular Health: Data from the Baltimore Longitudinal Study of Aging

**DOI:** 10.1371/journal.pone.0010099

**Published:** 2010-04-09

**Authors:** Erin Golden, Ana Emiliano, Stuart Maudsley, B. Gwen Windham, Olga D. Carlson, Josephine M. Egan, Ira Driscoll, Luigi Ferrucci, Bronwen Martin, Mark P. Mattson

**Affiliations:** 1 Cellular and Molecular Neurosciences Section, National Institute on Aging Intramural Research Program, Baltimore, Maryland, United States of America; 2 Metabolism Unit, National Institute on Aging Intramural Research Program, Baltimore, Maryland, United States of America; 3 Receptor Pharmacology Unit, National Institute on Aging Intramural Research Program, Baltimore, Maryland, United States of America; 4 Longitudinal Studies Section, Clinical Research Branch, National Institute on Aging Intramural Research Program, Baltimore, Maryland, United States of America; 5 Diabetes Section, National Institute on Aging Intramural Research Program, Baltimore, Maryland, United States of America; 6 Laboratory of Personality and Cognition, National Institute on Aging Intramural Research Program, Baltimore, Maryland, United States of America; Sapienza University of Rome, Italy

## Abstract

**Background:**

Besides its well-established role in nerve cell survival and adaptive plasticity, brain-derived neurotrophic factor (BDNF) is also involved in energy homeostasis and cardiovascular regulation. Although BDNF is present in the systemic circulation, it is unknown whether plasma BDNF correlates with circulating markers of dysregulated metabolism and an adverse cardiovascular profile.

**Methodology/Principal Findings:**

To determine whether circulating BDNF correlates with indices of metabolic and cardiovascular health, we measured plasma BDNF levels in 496 middle-age and elderly subjects (mean age ∼70), in the Baltimore Longitudinal Study of Aging. Linear regression analysis revealed that plasma BDNF is associated with risk factors for cardiovascular disease and metabolic syndrome, regardless of age. In females, BDNF was positively correlated with BMI, fat mass, diastolic blood pressure, total cholesterol, and LDL-cholesterol, and inversely correlated with folate. In males, BDNF was positively correlated with diastolic blood pressure, triglycerides, free thiiodo-thyronine (FT3), and bioavailable testosterone, and inversely correlated with sex-hormone binding globulin, and adiponectin.

**Conclusion/Significance:**

Plasma BDNF significantly correlates with multiple risk factors for metabolic syndrome and cardiovascular dysfunction. Whether BDNF contributes to the pathogenesis of these disorders or functions in adaptive responses to cellular stress (as occurs in the brain) remains to be determined.

## Introduction

The development, survival and plasticity of the vertebrate nervous system rely on the secretion of neurotrophins by neural cells. Brain-derived neurotrophic factor (BDNF) belongs to the neurotrophin family and exerts its actions by activating the tropomyosin-related kinase receptor B (TrkB) [1]. BDNF is involved in learning and memory formation [2] and reduced BDNF levels in various brain regions have been implicated in the pathogenesis of neurodegenerative and psychiatric disorders [3]. It has more recently become apparent that BDNF is present outside of the central nervous system (CNS) and circulates systemically [4], [5]. Studies using animal models have shown that conditions linked to metabolic and cardiovascular dysfunction, e.g. obesity, diabetes, heart disease, can be modified by manipulation of BDNF in the brain and in the peripheral circulation [6]. In rodents, it has been suggested that BDNF can cross the blood-brain barrier [7] and one study indicated that cortical levels of BDNF correlate with platelet BDNF concentration [8]. However, a recent study showed that BDNF concentration in the plasma is unrelated to levels found in the cortex and hippocampus [9].

Considerable evidence supporting a role for BDNF in energy homeostasis has been derived from experimental murine models. While homozygosity for the BDNF gene deletion is lethal [10], BDNF haploinsufficiency is associated with hyperphagia and obesity [6] and elevated endocrine appetite/dietary factors [11]. Consistent with data from BDNF heterozygotic mice, peripheral injection of BDNF causes a marked decrease in food intake and weight loss [12]. Reinforcing its role in peripheral and CNS metabolic control, BDNF and its receptor, TrkB, are abundantly expressed in hypothalamic areas associated with energy balance, such as the paraventricular nucleus, arcuate nucleus and ventromedial nucleus [13]. Moreover, BDNF deficiency in humans appears to induce phenotypes similar to those of animal models. Severe hyperphagia and childhood onset obesity develop in individuals with BDNF haploinsufficiency [14]. Furthermore, a de novo missense mutation of the gene that encodes TrkB is associated with childhood obesity [15].

While there is significant evidence suggesting a link between BDNF expression and energy regulation, the effects of BDNF on the cardiovascular system are not as well understood. BDNF is involved in the development and survival of the arterial baroreceptor system [16], and when injected in the rostrolateral medulla causes a blood pressure spike [17]. Additionally, embryonic BDNF deficiency severely impairs the development of intramyocardial vessels and can lead to cardiac hypocontractility [18]. Moreover, BDNF expression is significantly increased in atherosclerotic coronary arteries, compared to nonatherosclerotic coronary arteries from control subjects [19], and one study has demonstrated that there are reduced plasma BDNF levels in patients with acute coronary syndromes [20].

In spite of evidence from animal studies showing effects of BDNF on energy regulation and the cardiovascular system, little is known about BDNF plasma levels in human health and pathological states. In this study, we address this important issue, by measuring plasma BDNF levels in a cohort of healthy middle age and elderly subjects enrolled in the Baltimore Longitudinal Study of Aging (BLSA), and attempt to identify physiological and pathological parameters that may be correlated with plasma BDNF levels.

## Materials and Methods

### Subjects

The Baltimore Longitudinal Study of Aging (BLSA) is a prospective study of community-dwelling volunteers who were healthy at the time of enrollment; the study has been conducted by the National Institute on Aging without interruption since 1958 [21]. Institutional Review Board written approval was obtained from the National Institute on Aging, and informed written consent was obtained from all participants. All samples collected were analyzed in a de-identified manner. Participants returned to the National Institute on Aging Clinical Unit in Baltimore, Maryland, at regular intervals for 2–3 days of medical, physiological, and psychological examinations. Descriptive characteristics of the study cohort are reported in Table 1. Study participants with long-term chronic disease, severe allergies or un-controlled medical conditions were not included in the analytical study. Plasma BDNF levels were measured in 245 males and 251 females; genders were analyzed separately. This study was performed in an aged population; the average male was 71.9 years of age and the average age of the female cohort was 70.3 years of age. Blood samples were drawn from subjects in the morning after an overnight fast.

**Table 1:** Descriptive characteristics of study subjects.

Variable	Males	Females
Total	245	251
Mean age (years)	71.9±0.79	70.3±0.76
Weight (kg)	82.6±0.83	70.2±0.96
BMI (kg/m^2^)	27.0±0.25	26.9±0.35
Insulin use	3.2	0.44
Anti-depressant use	4.4	6.1
DM self-report	14.6	3.9
DM medication	15.1	5.2
Angina pectoris	15.9	5.7
Myocardial ischemia	4.9	3.9
Current smoker	4.8	2.6

Body Mass Index (BMI), Diabetes Mellitus (DM). Values for age, weight, and BMI are the mean ± S.E.M.

### Variables measured

#### Plasma BDNF:

Plasma BDNF levels were measured as described previously [22], using a commercially available ELISA kit (Promega) with the range of sensitivity from 7.8 to 500 pg/mL and inter-assay variation measured at 8.8% (low concentration), 2.9% (medium concentration) and 2.2% (high concentration). Briefly, blood samples were centrifuged at 3000 rpm for 30 minutes at 4°C. Plasma was carefully collected and was snap frozen on dry ice and subsequently stored at −80°C, until used for further analyses. For BDNF measurements, the plasma samples were diluted 1∶5 in block and sample buffer provided by the kit. The BDNF plate was coated with primary BDNF antibody overnight, the following day block and sample buffer was added to each well for 1 hour, and subsequently the standards and samples were added for 2 hours. Thereafter, anti-human BDNF pAb secondary antibody was added for 2 hours and the anti-Ig Y HRP conjugate was added for 1h. Then, TMB One solution was added to each well for 5 minutes and the reaction was stopped with hydrochloric acid. The plate was analyzed within 30 minutes, at 450 nm. All reagents necessary were provided by the manufacturer.

#### Blood pressure and body mass index:

Blood pressure (BP) determinations were performed in the morning, after a light breakfast, with participants in the seated position and following a 5-minute resting time. BP was measured three times in both arms with a mercury sphygmomanometer, and the average of the second and third measurements on both the right and left arms were used for the analyses. Height and weight were objectively measured and body mass index (BMI) was calculated as body weight (kg)/height (m^2^).

#### Fasting plasma lipids and glucose:

Plasma triglycerides and total cholesterol levels were determined by an enzymatic method (ABA-200 ATC Biochromatic Analyzer, Abott Laboratories). Low density lipoprotein (LDL) cholesterol was estimated by the Friedewald formula for those participants with triglycerides not greater than 400 mg/dL. Glucose levels were measured by the glucose oxidase method (Beckman Instruments Inc.).

#### Plasma hormone, sex hormone binding globulin, and folate levels:

Free triiodo-thyronine (FT3) levels were measured using a competitive-binding immunoassay (Beckman Coulter DX1800) by the Mayo Clinic Laboratories, as described previously [23]. Adiponectin was measured by radioimmuno assay, according to the manufacturer's instructions (Millipore). Bioavailable testosterone was determined by a modification of the ammonium sulfate precipitation method as previously described [24]. Sex hormone binding globulin (SHBG) was measured using an enzyme-linked immunosorbent assay using commercially available kits (DRG Diagnostics, Marburg, Germany). Free testosterone was calculated from total testosterone and SHBG by the formula of Vermeulen et al. [25]. Folate levels were determined by competitive protein-binding assays (Modular Analytics E170, Roche Diagnostics).

#### Statistical analyses:

Linear regression analysis, for individual subject plasma BDNF levels against the multiple parameters measured in the BLSA test population, was performed for all test subjects whose BDNF levels fell within a normal distribution (male and female separately) for the total BDNF measurements. This exclusion criteria (95% confidence) resulted in the removal of four subjects from both male or female BLSA subject groups. Standard linear regression analyses were performed using GraphPad Prism (version 3). Statistical significance was taken at the 0.05 level with respect to the linear correlations' deviation from a zero slope. Linear regression correlation analysis was performed for plasma BDNF levels versus all of the parameters measured in BLSA. Linear correlations that failed to pass the cut-off criteria of p<0.05 are listed in Supplementary Table S1.

## Results

### Plasma BDNF, gender, and age

**Figure 1:**
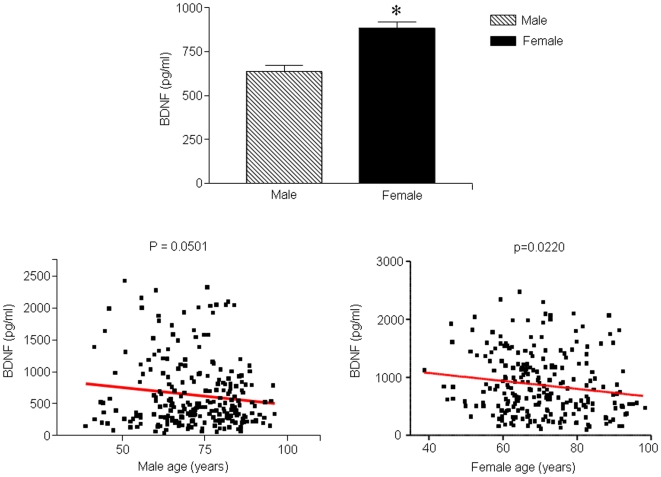
Plasma BDNF, gender and age. A. Plasma BDNF was significantly higher in females than in males (p<0.05). B and C. Multiple linear regression revealed that plasma BDNF was inversely correlated with age in males (p = 0.0501, R^2^ = 0.01577) and in females (p = 0.0220, R^2^ = 0.02244).

Across the two gender populations in our study the plasma BDNF levels were significantly higher in female subjects than in males (p<0.05; Figure 1A). In both male and subjects, a significant negative correlation was seen between BDNF and age, with older males and females having lower plasma BDNF levels (males: p = 0.0501, R^2^ = 0.0157; females: p = 0.022, R^2^ = 0.02244; Fig. 1B, C, Table 2).

**Table 2:** Linear Regression analysis of plasma BDNF and metabolic and cardiovascular dysfunction risk factors, measured in males and females in the BLSA cohort.

Variable	p-value	F value	DFn, DFd	R^2^
**Male**				
Diastolic blood pressure	0.0300	4.765	1.000, 237.0	0.01971
Triglycerides	0.0289	4.834	1.000, 235.0	0.02016
Bioavailable Testosterone	0.0443	4.092	1.000, 229.0	0.01755
SHBG	0.0150	6.037	1.000, 167.0	0.03489
FT3	0.0053	7.932	1.000, 229.0	0.03348
Age	0.0501	3.878	1.000, 242.0	0.01577
Adiponectin	0.0137	6.242	1.000, 128.0	0.04650
Glucose-120	0.0460	4.037	1.000, 182.0	0.02170
**Female**				
Diastolic blood pressure	0.0486	3.930	1.000, 232.0	0.01666
LDL	0.0139	6.142	1.000, 227.0	0.02634
Cholesterol	0.0040	8.455	1.000, 234.0	0.03487
Folate	0.0396	4.285	1.000, 223.0	0.01885
Fat mass	0.0490	3.922	1.000, 211.0	0.01825
BMI	0.0396	4.282	1.000, 228.0	0.01843

SHBG, sex-hormone binding globulin; FT3, Free triiodo-thyronine T3; LDL, low density lipoprotein; BMI, bodymass index.

### Elevated BDNF levels are correlated with risk factors for heart disease and metabolic syndrome in both male and female subjects

**Figure 2:**
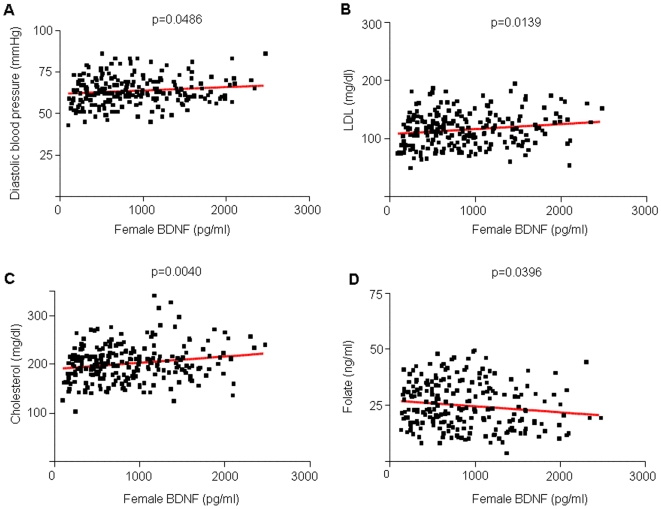
Linear regression analysis correlating plasma BDNF and markers of cardiovascular risk in females. A, B and C. Plasma BDNF was positively correlated with diastolic blood pressure (p<0.05, R^2^ = 0.0228), LDL-cholesterol (p = 0.139, R^2^ = 0.02634), and total cholesterol (p = 0.0040, R^2^ = 0.03487). D. Folate levels were inversely correlated with plasma BDNF (p = 0.0396, R^2^ = 0.01885).

**Figure 3:**
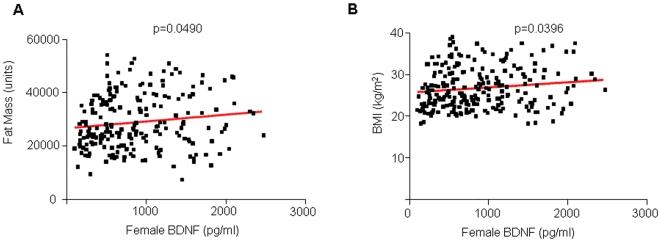
Linear regression analysis of plasma BDNF, fat mass and BMI in females. A and B. Plasma BDNF was positively correlated with fat mass (p = 0.0490, R^2^ = 0.01825) and BMI (p = 0.0396, R^2^ = 0.01843).

**Figure 4:**
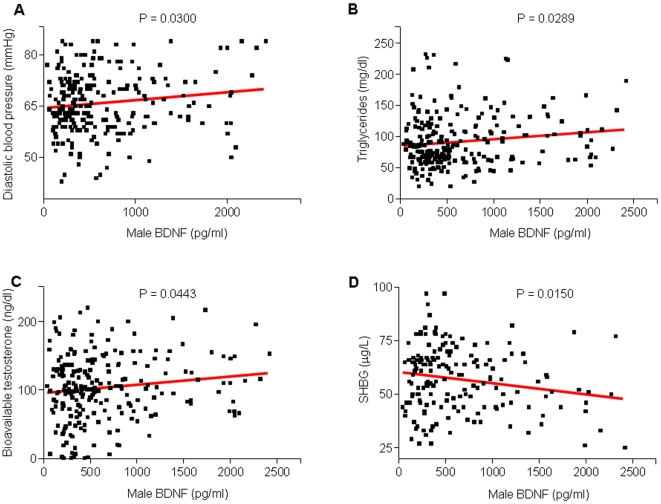
Linear regression analysis correlating plasma BDNF and markers of the metabolic syndrome in males. A, B and C. Plasma BDNF was positively correlated with diastolic blood pressure (p = 0.0300, R^2^ = 0.01971), triglycerides (p = 0.0289, R^2^ = 0.02016), and bioavailable testosterone (p = 0.0443, R^2^ = 0.01755). D. Plasma BDNF was inversely correlated with SHBG (p = 0.0150, R^2^ = 0.03489).

Although the exact nature of the relationship between BDNF and heart disease is unknown, several studies have suggested that increased BDNF levels are associated with risks for coronary heart disease [26]–[28]. BDNF is expressed in smooth muscle cells, macrophages and extracellular matrix of diseased cardiac tissue [19] and pretreatment with BDNF can lead to increased myocardial injury in a rat model of myocardial infarction [29]. Our results further support the association between BDNF levels and cardiac disease. In females, increased BDNF levels were associated with increased diastolic blood pressure (p = 0.0486, R^2^ = 0.01666), increased LDL (p = 0.0139, R^2^ = 0.02634), increased cholesterol (p = 0.0040, R^2^ = 0.03487), increased fat mass (p = 0.0490, R^2^ = 0.01825) and increased BMI (p = 0.0396, R^2^ = 0.01843; Figure 2 and Figure 3). In males, increased BDNF levels were associated with increased diastolic blood pressure (p = 0.03, R^2^ = 0.0197) and increased triglycerides (p = 0.0289, R^2^ = 0.02016; Figure 4A, B, Table 2). The association of these risk factors with increased BDNF levels could suggest the presence of either a reactive protective role of BDNF in response to the pathophysiology or a contributory role in the etiology of the disease itself.

### Plasma BDNF and folate

BDNF was negatively correlated with folate levels in female subjects (p = 0.0396, R^2^ = 0.01885, Figure 2D, Table 2). Folate, also referred to as folic acid or vitamin B9, is essential for red blood cell production and the prevention of anemia. Additionally, folate is needed to prevent the accumulation of homocysteine.

### BDNF is associated with sex hormone levels in males

In male subjects we observed significant associations of BDNF with bioavailable testosterone (BT) and sex hormone binding globulin (SHBG). There was a significant positive correlation between BDNF and BT; males with higher BDNF levels tended to have more BT (p = 0.0443, R^2^ = 0.01755; Figure 4C, Table 2). The majority of circulating testosterone in the body is bound to SHBG, thus one would expect to see opposite changes in levels of these two hormones. Indeed, in our study subjects, males with higher BDNF tended to have lower SHBG (p = 0.0150, R^2^ = 0.03489; Figure 4D, Table 2). Typically during aging, BT tends to decrease while SHBG increases [30].

### BDNF is associated with multiple metabolic hormones in males

**Figure 5:**
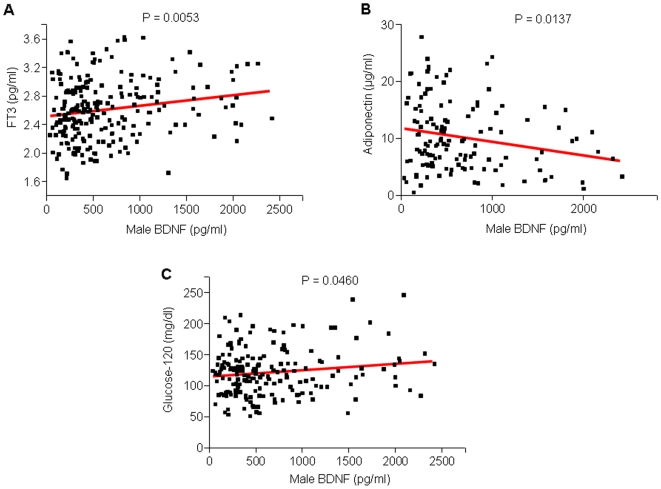
Linear regression analysis correlating plasma BDNF with thyroid function, adiponectin levels and glucose sensitivity in males. A and C. Plasma BDNF was positively correlated with FT3 (p = 0.0053, R^2^ = 0.03348) and glucose-120 (p = 0.0460, R^2^ = 0.02170). B. Plasma BDNF was inversely correlated with plasma adiponectin (p = 0.0137, R^2^ = 0.04650).

Adiponectin is an adipose derived hormone that modulates glucose regulation and fatty acid catabolism. We observed a significant negative correlation between BDNF and adiponectin in male subjects (p = 0.0137, R^2^ = 0.0465). Males with higher plasma BDNF tended to have lower plasma adiponectin levels (Figure 5B, Table 2). A positive correlation was seen between BDNF and free triiodo-thyronine (FT3) in male subjects (p = 0.0053, R^2^ = 0.03348; Figure 5A, Table 2).

### Plasma BDNF and glycemic sensitivity

In our aged individual samples, a positive correlation was demonstrated between male BDNF plasma levels and glucose levels 120 minutes after an oral glucose bolus (p = 0.0460, R^2^ = 0.02170), further suggesting a role for BDNF in metabolic function (Figure 5C, Table 2). However, we did not find a correlation between fasting plasma glucose and plasma BDNF.

### Variables measured, but not significantly correlated with plasma BDNF levels

In addition to the factors measured that did exhibit significant correlations with plasma BDNF levels in males and/or females, we also measured a range of additional factors for which no statistically significant correlation with BDNF levels was obtained in either males or females (Supplementary Table S1). These included HDL, cholesterol, vitamin B12, ferritin, total iron, homocysteine, uric acid, lactate dehydrogenase, body weight, fasting glucose, resistin, FT4, T4, TSH and total testosterone. We also normalized our data set for body mass index (BMI), to further determine relationships between BDNF and cardiovascular and metabolic factors. BMI is considered to be an imperfect measure because it does not assess body fatness directly [31]. Due to this, its use in epidemiologic studies has been recently criticized [32]–[34]. It has been demonstrated that for example with respect to a profoundly declarative outcome such as mortality, that weak or negative associations between BMI and mortality are due to the lack of the ability of BMI to discriminate between lean and fat mass and that more accurate and nuanced assessments of body-type are required [35]. Indeed the predictive capacity of long-term health outcomes using BMI-rankings can skew data and result in misleading findings [36]. When normalizing for BMI, in females plasma BNDF levels were negatively correlated with leptin (p = 0.0790) and uric acid (p = 0.020) and in males, BDNF was negatively correlated with age (p = 0.055) and positively correlated with weight and uric acid levels (p = 0.079, p = 0.063 respectively). It is presently unclear which factors, either directly or indirectly, interact with BDNF and further work is needed to elucidate the complex metabolic endocrine networks that involve or affect BNDF.

## Discussion

The neurotrophic factor, BDNF, has in recent years been the subject of considerable interest primarily for its roles in developmental and synaptic plasticity, and as a neuroprotective factor for multiple aging-related neurodegenerative disorders [3]. However, evidence is scant regarding the function of BDNF outside of the CNS. Our data from middle age and elderly subjects in the BLSA demonstrated that plasma BDNF levels are significantly associated with several key indicators of metabolic and cardiovascular health. These results reinforce an increasing body of evidence indicating regulatory roles for BDNF in metabolism and cardiovascular homeostasis. Higher plasma BDNF levels were associated with risk factors for cardiovascular disease including elevated diastolic blood pressure and triglycerides in men and elevated diastolic blood pressure, total and LDL cholesterol, BMI and fat mass in women. Although the correlations reported in our study are robust, we cannot derive any conclusions on the specific nature of these associations. It is possible that BDNF contributes to the pathophysiology of cardiovascular disease, or elevated plasma BDNF may represent a compensatory response to an underlying disease processes.

We found that plasma BDNF levels decrease with age in both males and females, which is similar to what has been reported previously [37]. The specific source of plasma BDNF is unknown. In addition to the central and peripheral nervous system, BDNF is also stored in circulating platelets and is found in muscle, heart and gonads [19], [38]–[40]. The exact reason for a decrease in plasma BDNF levels with aging is unclear. Considering that the prevalence of metabolic syndrome, type 2 diabetes, obesity, coronary artery disease and hypertension increases as individuals get older, it is possible that BDNF functions as a protective factor against metabolic and cardiovascular disorders, and that decreased peripheral BDNF synthesis, secondary to aging, renders individuals more susceptible to these conditions. In regards to metabolic dysfunction and aging, it has been suggested that the connection between energy regulation and increasing age may also be controlled by alterations in BDNF levels [41]. Furthermore, low plasma BDNF levels may be linked to increased mortality. Data from the Danish National Register of Patients indicated a significantly greater all-cause mortality risk in elderly women with low plasma BDNF levels, independently of education, CNS disease, cardiovascular disease, cancer, respiratory disease and low-grade inflammation [42].

Similarly to the sex-differences that have been described for leptin [43], we found that plasma BDNF levels were significantly higher in women than in men. Additionally, we also found that plasma BDNF levels correlated positively with BMI and fat mass in women, but not in men. Unlike leptin, however, BDNF has not been shown to be secreted by adipocytes. Recently, a small study of 18 women undergoing bariatric surgery followed by successful weight loss showed that plasma BDNF levels were significantly decreased 3 months postoperatively [44], which corroborates our finding of a positive correlation between BMI and plasma BDNF levels. The higher levels of plasma BDNF associated with an increased fat mass could potentially reflect a systemic low-grade inflammatory state. It has recently been demonstrated that even non-obese individuals with moderate abdominal adiposity have higher plasma C-reactive protein (CRP) levels than BMI-matched controls [45]. As proinflammatory cytokines can stimulate BDNF secretion from monocytes, the elevated plasma BDNF levels could indicate an inflammatory state associated with greater adiposity [46]. Moreover, we found that plasma adiponectin was inversely correlated with plasma BDNF levels in males. Rodent and human studies show that adiponectin decreases in conditions of metabolic stress such as obesity and type-2 diabetes, while weight loss increases plasma adiponectin levels [47]. For the female subjects, the data was not corrected for estrogen replacement therapy due to lack of information and reliable data on estrogen replacement status in the BLSA. Future studies are needed to elucidate these relationships further.

Another known risk factor for metabolic syndrome, type 2 diabetes and coronary artery disease is male hypogonadism. In addition, decreased levels of plasma SHBG have also been implicated in both insulin resistance and metabolic syndrome [37]. It has also been proposed that SHBG may act as a regulator of lipid metabolism [48]. We found that bioavailable testosterone levels were directly correlated to plasma BDNF levels in males, and the opposite occurred for SHBG, indicating that plasma BDNF could play a role in the pathogenesis of metabolic and cardiovascular disorders in male hypogonadism. In males, we also found that levels of the unbound form of the thyroid hormone triiodothyronine (T3) were positively correlated with plasma BDNF levels. T3 is an orexigenic hormone that plays an important role in metabolic regulation [49]. T3 has various peripheral metabolic actions, which include regulation of hepatic gluconeogenesis, lipogenesis, and glucose transporter expression in skeletal muscle [50]. A recent study demonstrated that T3 is involved in the regulation of BDNF gene expression in hypothalamic areas involved in energy balance [51], indicating a potential interface between T3 and BDNF. It has also been shown that treatment with T3 can increase the expression of hypothalamic neuropeptide Y (NPY) mRNA, while reducing hypothalamic pro-opiomelanocortin (POMC) and cocaine and amphetamine-regulated transcript (CART) mRNA levels [52]. NPY, CART and POMC are satiety factors, and NPY is known to regulate energy balance, food intake and physical activity. CART has been closely associated with the action of two important regulators of food intake, leptin and NPY [53].

The significant correlation between plasma BDNF, plasma lipids and diastolic blood pressure in both males and females strongly suggests that plasma BDNF is important for cardiovascular health. In addition to potentially playing a direct role in atherogenesis, plasma BDNF may be a regulator of lipid metabolism and blood pressure control. Dislipidemia and hypertension are major risk factors for coronary heart disease. Whether the associations described above are causal or whether elevated plasma BDNF represents a compensatory response to disrupted lipid metabolism and hypertension is presently unclear. It remains to be elucidated if the link between dislipidemia and BDNF is the presence of an inflammatory state. Intriguingly, in our study, plasma folic acid levels were inversely correlated with plasma BDNF levels in women. Folate deficiency is the most common nutritional cause of hyperhomocysteinemia, a well-established risk factor for atherogenesis and thrombosis [54].

The relationship between BDNF and glucose regulation is intricate. We found that the 2-hour blood glucose time-point, following an oral glucose load, was positively correlated with plasma BDNF levels. It has been shown recently that plasma BDNF levels are reduced in patients with type 2 diabetes (independently of obesity), and are inversely correlated with fasting plasma glucose [55]. Additionally, serum levels of BDNF have also been shown to be decreased in patients with type 2 diabetes compared to healthy controls [56].

In complex biological systems, coordinated metabolic functions are created by the summation of multiple inter-connected pathways forming networks of varying sizes and relative importance [57]–[59]. Thus the ability to apply significance of predicted functional output no longer rests upon individual factors but on co-presentation and coherent regulation of these factors, reflecting the coordinated, interconnected nature of metabolic pathways themselves. It is highly likely that a simple resultant physiologically-measured parameter, such as blood pressure or serum triglycerides, can be related to small, often non-statistically significant, coherent actions of multiple factors that together generate the measured effect. All the factors that we measured typically fell into two functional categories, metabolic factors and cardiovascular factors. Thus, even though the statistical significance of the correction of each factor individually with BDNF is often small, all those factors acting synergistically together will likely elicit a significant biological phenotype. This concept of inter-connectivity is currently being applied in genomic analyses, where often small, non-significant gene changes that occur together can elicit large clinically-relevant phenotypic outputs [60]–[64]. In future studies, this idea of connectivity will also likely be applied to clinical studies.

The aging process and many of its associated diseases all involve perturbed energy metabolism. The control of food intake, glucose regulation and metabolism is dependent upon a fine balance between central regulatory inputs (primarily orchestrated by the hypothalamus), and a multitude of peripheral signals, such as insulin, adipokines (e.g. leptin, adiponectin, resistin), and gut hormones (e.g. cholecystokinin and ghrelin). The crosstalk between these myriad pathways subserves the control of energy balance, stress responses and cardiovascular function. It is becoming apparent that BDNF plays an important regulatory role within this complex and dynamic system, and that the specific roles of BDNF in controlling and maintaining peripheral metabolic and cardiovascular health require further investigation. Alterations in peripheral BDNF signaling could potentially be a common denominator for the metabolic syndrome spectrum, which ranges from impaired glucose tolerance to overt diabetes, to mild atherogenesis to clinical coronary artery disease. Gaining a greater understanding of plasma BDNF's potential multifaceted roles in both the periphery and the CNS will be fundamental for advancing our knowledge of mechanisms underlying metabolic disorders and for the development of novel therapies that can modify BDNF levels in specific target tissues.

## Supporting Information

Table S1 Linear Regression analysis of plasma BDNF and metabolic and cardiovascular dysfunction risk factors, measured in males and females in the BLSA cohort.
(0.05 MB DOC)
