# Supplementary material for: Circulating Brain-Derived Neurotrophic Factor and Indices of Metabolic and Cardiovascular Health: Data from the Baltimore Longitudinal Study of Aging
Source: PLoS One. 2010 Apr 9;5(4):e10099. doi: 10.1371/journal.pone.0010099 (PMC2852401; doi:10.1371/journal.pone.0010099)
Supplement: Table S1 — Linear Regression analysis of plasma BDNF and metabolic and cardiovascular dysfunction risk factors, measured in males and females in the BLSA cohort. (0.05 MB DOC) [file pone.0010099.s001.doc]

**Supplementary Table S1.** Linear Regression analysis of plasma BDNF and metabolic and cardiovascular dysfunction risk factors, measured in males and females in the BLSA cohort.

| **Variable** | **p value**  **Males Females** | |
| --- | --- | --- |
| Age | 0.05 | 0.02 |
| Triglycerides | 0.03 | 0.32 |
| Cholesterol | 0.10 | 0.004 |
| LDL | 0.20 | 0.01 |
| HDL | 0.87 | 0.97 |
| Folate | 0.80 | 0.04 |
| Vitamin B12 | 0.92 | 0.39 |
| Ferritin | 0.98 | 0.27 |
| Iron | 0.53 | 0.51 |
| Homocysteine | 0.49 | 0.10 |
| Uric acid | 0.93 | 0.10 |
| LDH | 0.74 | 0.60 |
| Weight | 0.28 | 0.23 |
| Fat mass | 0.29 | 0.05 |
| BMI | 0.18 | 0.04 |
| Glucose 0 | 0.31 | 0.87 |
| Glucose 120 | 0.05 | 0.82 |
| Adiponectin | 0.01 | 0.65 |
| Leptin | 0.89 | 0.06 |
| Resistin | 0.38 | 0.24 |
| FT3 | 0.005 | 0.11 |
| FT4 | 0.27 | 0.87 |
| T4 | 0.67 | 0.96 |
| TSH | 0.94 | 0.70 |
| Total T | 0.78 | 0.38 |
| Bioavailable T | 0.04 | 0.67 |
| SHBG | 0.01 | 0.07 |
| Estradiol | 0.65 | 0.06 |

High density lipoprotein (HDL); Free triiodo-thyronine T4 (FT4); Triiodo-thyronine 4 (T4); Testosterone (T); Thyroid Stimulating Hormone (TSH); Lactate Dehydrogenase (LDH).
